# Supplementary material for: Are carbon emissions trading and green financial instruments synergistic? -Comprehensive quantitative research based on content analysis
Source: PLoS One. 2024 Mar 7;19(3):e0298601. doi: 10.1371/journal.pone.0298601 (PMC10919601; doi:10.1371/journal.pone.0298601)
Supplement: S1 Data — (ZIP) [file pone.0298601.s001.zip › Data/地级市Z_F工作报告.zip!/╜¡╬≈/╔╧╚─╩╨/└┤╫╘╣1⁄2╓┌║┼í╛┬φ┐╦╩2╛▌═°í┐_2007.docx]

各位代表：

　　现在，我代表市人民政府向大会作工作报告，请予审议，并请市政协各位委员和列席会议的同志提出意见。

　　一、2006年工作简要回顾

　　过去的一年，是“十一五”的开局之年，也是上饶继往开来、乘势而上、科学发展的一年。在省委、省政府和市委的正确领导下，全市上下以邓小平理论和“三个代表”重要思想为指导，认真落实科学发展观，深入贯彻国家加强宏观调控的各项政策措施，以创新创优创先的精神、敢想敢为敢闯的干劲，全力实施“十一五”规划，圆满完成市一届人大十次会议确定的各项目标任务，实现了“十一五”的良好开局。

　　——经济总量不断做大。全市实现生产总值451.4亿元，增长13.6%，比全省平均水平快1.3个百分点；实现财政总收入41亿元，增长22.2%，比全省平均水平快0.5个百分点；完成全社会固定资产投资318.3亿元，增长39.3％，超出全省平均水平15.6个百分点，其中城镇以上固定资产投资247.4亿元，同比增长38.7%；完成社会消费品零售总额158亿元，增长16.4%，增幅居全省第三位，比全省平均水平快0.9个百分点。实际利用市外资金284亿元，增长32.2%；实际利用外资1.86亿美元，增长16.5%，超出全省平均水平0.6个百分点；外贸出口1.65亿美元，增长29%。12个县（市、区）财政收入全部超过亿元，总量达到36.9亿元，占全市财政总收入的90％，过千万元的乡镇达到44个，比上年增加13个。

　　——增长质量不断提高。财政收入占GDP比重为9.1%，提高0.4个百分点，税收收入占财政总收入的74.5%，提高3个百分点；全部工业占GDP的比重为33.3%，提高2.3个百分点；非公有制经济增加值占GDP比重为66.7%，提高6.5个百分点；全社会固定资产投资增幅比上年回落2.4个百分点，其中完成工业投资99.4亿元，增长53.8％，占城镇以上固定资产投资的40.2%，增长6％。

　　——产业结构不断优化。三次产业比重为19.4：44.5：36.1，同比一产下降1.8个百分点，二产上升2.0个百分点，三产下降0.2个百分点。完成一产增加值87.7亿元，增长6.0%，粮食总产达到53.3亿斤，增长1.7％；实现全部工业增加值150.1亿元，增长18.5％；全市园区工业企业实现主营业务收入215.3亿元，增长54％，实现税金13.1亿元，增长43.9％；三产实现增加值163亿元，增长14.3％。

　　——城市品位不断提升。全市投入城市基础设施建设资金65亿元，实施了一批重点城建工程，新建改造绿地面积252. 8 万平方米 。上饶市创建“中国优秀旅游城市”获得成功，广丰、玉山、万年、婺源、上饶县等5个县城被评为省园林城市。全市城镇化率比上年提高2.5个百分点，达到29.5%。

　　——基础设施不断加强。全市境内高速公路总里程达到 288.5公里 ，农村公路完成路面硬化 1800公里 。黄金埠电厂一期60万千瓦时发电机组投产运行；新建和改造22万伏变电站3座、11万伏变电站2座，配套新建供电线路 217公里 。完成病险水库除险加固332座，占总数的42.4%。

　　——人民生活不断改善。农民人均纯收入达到3524元，增长9.2％，市区城镇居民人均可支配收入达到9832元，超过全省平均值281元，增长14％，比全省平均水平快3.2个百分点。全市各金融机构人民币存款达453.8亿元，贷款为324.7亿元，存贷款总额分别在全省排第三和第二位。存款的增幅为19.2%，超全省平均水平1.5个百分点。

　　——社会事业不断进步。以低保、养老保险、医疗保险、困难群众救助为主的社会保障体系初步建立，教育事业得到巩固和加强，公共卫生事业取得新进展，机构编制和人事人才工作稳步推进，文化、广电、体育事业日趋繁荣，国家、省级科技立项创造历史最好成绩，人口与计划生育工作成效明显，绿色生态上饶建设进展顺利，普法教育深入开展，社会矛盾排查调处机制、突发性公共事件应急处理机制和社会治安防控体系逐步建立，安全生产形势总体趋于平稳，社会保持和谐稳定。新闻出版、地方志、档案、保密、民族、宗教、外事侨务、涉台事务、气象服务、防震减灾、民兵预备役和“双拥”工作、国防教育、人民防空、社会福利、妇女儿童、老龄、残疾人、慈善等事业健康发展。

　　各位代表，过去一年的成就来之不易。上饶发展取得的每一个成绩，政府工作取得的每一点进步，都是省委省政府和市委正确领导的结果，都凝聚着全市人民的智慧和力量，都得益于全市人民的理解和支持。在此，我谨代表市人民政府，向全市人民和驻饶人民解放军、武警官兵、公安干警和中央、省驻饶企业，向给予政府工作支持和监督的人大代表、政协委员、各民主党派、工商联、无党派人士和各人民团体，向关心和支持上饶现代化建设的海内外朋友，向广大老干部、老同志和关心家乡发展的在外乡友，在上饶创业的境内外投资者、合作者，表示衷心的感谢！

　　在肯定成绩的同时，我们也清醒地看到在经济社会发展中还面临一些矛盾和问题，主要是：经济结构调整的任务还很重，企业自主创新能力还比较弱，现代服务业发展水平还不高，“三农”问题仍然比较突出，就业和社会保障压力较大，有少部分群众生活还比较困难，政府社会管理、公共服务水平还有待进一步提高，环境保护、食品药品安全、安全生产和社会治安等领域还存在不少问题。对上述问题，我们将采取有力措施，认真加以解决。

　　二、2007年工作目标和主要措施

　　今年政府工作的总体要求是：以邓小平理论、“三个代表”重要思想和科学发展观为指导，全面贯彻落实市第二次党代会精神，围绕“建设四省交界区域中心城市和快速发展地区”这一目标，把握又好又快发展这一主题，切实转变经济增长方式,深入推进改革开放，努力创新政府管理，不断提高人民群众的幸福感，为建设一个富强民主文明和谐的新上饶奠定坚实基础。

　　综合考虑各种因素，2007年全市经济社会发展的总体目标是：生产总值增长15%，达到528亿元；财政总收入增长22%，达到50.1亿元；全社会固定资产投资增长27.2%，达到405亿元；社会消费品零售总额增长14.6%，达到181亿元；城区居民人均可支配收入增长10%，达到10815元，农民人均纯收入增长6.7%，达到3760元；城市化率达到31.5％；城镇登记失业率控制在3.8%以内；消费品零售价格指数控制在102%以内；万元生产总值综合能耗下降4%；主要污染物排放总量减少2%；人口自然增长率控制在8.05‰以内。

　　为实现上述目标，要着重抓好以下工作：

　　（一）着力促进经济社会又好又快发展

　　切实把科学发展观贯穿于经济社会发展的全过程，落实到经济社会发展的各个环节,推动经济社会发展真正转入以人为本、全面协调可持续发展的轨道,实现经济社会又好又快发展。

　　第一，以发展现代农业为重点，加快建设社会主义新农村。

　　切实抓好粮食生产。继续执行粮食最低收购价政策，及时发放各种粮食补贴，调动农民种粮积极性，确保今年粮食总产保持在52亿斤以上。加大农业产业结构调整力度，努力提高高效经济作物和养殖业的比重，积极推进“一村一品”工程。不断增强农业综合生产能力。开工建设伦潭水利枢纽，续建鄱阳沿河圩、余干枫富联圩、康山蓄滞洪区等防洪工程，继续实施好城市防洪二期工程、灌区的渠系配套和节水改造工程，以及余干、万年两个大型机电泵站更新改造工程，全面完成病险水库除险加固、240平方公里的水土流失治理，全力实施“造地增粮富民工程”，进一步加强农业科技服务体系建设。加快推进农业产业化经营。加速壮大农业龙头企业，力争新增国家级龙头企业1家，省级10家，市级40家，销售收入5000万元以上的龙头企业达到15家，1亿元以上的10家，10亿元以上的1家。力争已列入省“十百千亿工程”的春源绿色食品有限公司主营业务收入达到1.5亿元，澳门南光谷丰肉联加工厂产值过亿元；加快建设五洲星食品加工、优质农产品基地和大鄣山有机茶改造等项目，积极推进农业标准化生产。加快农产品市场体系建设，重视培育农村经纪人、农产品运销专业户和流通服务组织，培育多元化、多层次的市场流通主体。扎实推进社会主义新农村建设。按照“五让一规范”的要求，抓好1550个试点村的产业发展和村庄整治工作，夯实试点村产业发展基础，加快推进试点村饮水安全、道路改造、公共卫生、能源、信息化等工程，使试点村面貌有较大改观。

　　第二，以园区为重点，扎实推进新型工业化。

　　突出重点，促进工业园区快速发展。紧紧抓住入选全省“十百千亿工程”的契机，进一步加大工业园区建设力度，把上饶经济开发区和广丰、余干、玉山工业园区，逐步打造成为引领全市园区快速发展的引擎。建立和完善联席会议、协调会议制度，把开发区建设成为工业新城、城市新区，力争实现主营业务收入100亿元，利税总额10亿元；广丰工业园区实现主营业务收入105亿元，利税6亿元；余干工业园区实现主营业务收入30亿元，利税总额3亿元；玉山工业园区主营业务实现收入25亿元，利税总额1亿元:其它工业园区要进一步加大基础设施投入，创造条件，迎头赶上。

　　整合资源，促进产业快速集聚。坚持把优化生产力发展布局作为牵动全市经济发展的战略突破口，着力构筑“三大产业带”。一是精心构筑信江河谷产业带。重点打造新型建材产业、铜材加工、光学仪器制造产业、中高档客车制造产业、普通轴承加工食品加工、纺织服装产业基地，逐步把信江河谷产业带建设成江西省对接长三角的“桥头堡”和具有明显优势的先进制造业基地。二是精心构筑景婺黄（常）产业带。充分发挥“中国铜都”和“最美的乡村”的品牌效应，打造有色金属采冶及精深加工产业、以电子及生物制药为主的环保产业基地。三是精心构筑滨湖产业带。依托水面和土地资源优势，充分发挥黄金埠电厂等国家大型企业的扩散功能，加快建设电力产业、服装出口加工、机械制造产业和食品加工产业基地。

　　分类引导，促进骨干企业快速壮大。继续加大对全市50户重点调度骨干企业的扶持力度。加快推动福丰氟化盐、瓮福化工年产40万吨硫酸等已引进并投产项目进入正常生产运营。抓好已经列入国家级资源综合利用项目的华丰铜业年处理6万吨污泥综合利用项目、丰厦墙材的加气混泥土砌块项目，已上报国家发改委节能备选项目的德兴金山金矿采选电机系统节能工程，已经列入省级重大建设项目的弋阳海螺日产4500吨水泥项目、华玉矿业的长石精矿项目等新增项目，以及经筛选确定的50户市级重点投资项目的实施，努力培植推动地方经济发展的骨干力量。逐步形成以信江实业、华丰铜业、精元电脑、弋阳江冶、月兔丰华彩印、江西中都贵金属冶炼等主营业务收入超5亿元的企业为龙头，以江西广厦包装、广丰联晖实业、万年皇阳贡米、德兴百勤异VC钠、金山金矿、玉山万年青水泥、横峰繁荣铜业等一批主营业务收入超1亿元企业为支撑的梯次发展新格局。

　　第三，以商贸旅游为重点，促进现代服务业快速发展。

　　积极发展商贸物流业。启动城东区物流中心开发项目，加快浙江春和集团在上饶投资、合作项目和赣东北农产品批发大市场检验检测及信息化系统工程，抓紧江西上饶运通公司项目建设；加快新华龙现代物流园续建、汽车配件市场、二手车交易市场和报废车拆解中心项目建设。同时，抓好江南米业市场、万年珍珠市场扩建和鄱阳水产品批发市场续建工程。继续实施“万村千乡”工程，加快建设新农村现代流通服务网络工程，建设农家店800-1000家、上规模的配送中心2-3个。力争全市年营业额超亿元的大型综合商场（超市）达到7家；年交易额超亿元区域性重点综合专业市场达到21个，年营业额超亿元的特色商业街达到12条，主营收入超亿元的商贸流通服务龙头企业达到30户，限额以上连锁企业达到15个。

　　继续壮大旅游休闲业。充分发挥“中国优秀旅游城市”的品牌效应，加快旅游产品由单一观光型向观光度假型转变，旅游产业从单一数量型向数量质量并重型转变，旅游客源地由国内市场为主向国内国际市场并重转变。积极争创 5A 级景区。举全市之力做好三清山申遗工作，丰富三清山旅游的文化内涵，加快三清山金沙索道、金沙服务区建设，力争三清山旅游产业集团主营业务收入达到3亿元，利税总额过亿元。加快组建婺源旅游集团。加强红色旅游基地建设，加快推进上饶集中营旧址、方志敏革命根据地旧址风景名胜区改造工程，挖掘和开发葛仙山、黄岗山、铜钹山等旅游资源，做好龟峰丹霞地貌捆绑申遗工作。加强鄱阳湖旅游资源开发，加快发展滨湖旅游产业。

　　大力发展科技信息、金融保险、法律咨询等服务产业。努力做大做强市商业银行。加快物业管理、家政服务、职业介绍等社区服务业，体育健身、文化娱乐、宾馆餐饮等产业的发展步伐。

　　第四，以增强中心城市带动功能为重点，促进区域经济协调发展。

　　不断完善中心城市功能。力争投入城建资金131.7亿元，其中市本级中心城区投入33.7亿元，同比分别增长102%和60%，安排建设项目44个，其中续建项目19个，新开工项目25个，各县（市）完成投资80亿元。在城市道路交通方面:加快老城区庆丰路、茶圣路、铁路既有线改造，完成上饶东互通道路改造；完成中心区凤凰西路、府西路、府北路、龟峰大道、凤凰大桥续建工程，加快续建广信大道；完成水南片区丰溪路改造续建工程；完成城东片区货场路续建和三清山大道东延伸段建设，加快建设站前南路东西延伸段和凤凰大道东段续建；完成三江片区叶挺大道续建工程和茅家岭至周田旅游公路建设；完成旭日片区凤凰西路（上饶县段）续建工程；完成开发区上二路、上三路和饶二路建设。在功能配套方面，加快建设老城区中心广场商贸城和上饶救助中心；完成中心区市图书馆续建工程，加快建设奥林匹克中心；完成三江片区上饶卫校新校区建设。在民生设施方面，加快老城区紫阳公园续建；完成中心区双塔公园二期和中央公园续建工程，加快建设龙潭公园；加快水南片区信江书院改造一期和云碧峰国家森林公园二期工程建设，完成生活垃圾处理厂续建工程；加快城东片区20万吨自来水厂建设；加快开发区污水处理工程建设；加快建设中心城区10座垃圾中转站。同时完成40条里弄小巷改造和经济适用住房建设，以及市区居民饮用水安全综合整治工程和中心城区排污管网改造工程建设。

　　强力打造城市产业。以经济开发区为平台，重点打造制造业集聚基地。通过引进先进技术和生产工艺，发展科技含量高、关联度高、产业链长的项目，改造提升传统产业，积极推进有色金属、机械制造、纺织服装等主导产业上档升级。大力发展中心城区现代商贸物流业。加快春和集团大型超市项目、中心广场大型零售项目和汽车经济服务中心的建设。

　　提升城市管理水平。继续推进“五城”同创，完善创建工作标准体系、责任体系、监督体系和服务体系，进一步强化宣传教育，提高市民的道德素养，充分调动各方面积极性，形成城市管理的合力，把城市管理纳入科学规范、运转有序的轨道。

　　大力发展县域经济。进一步落实简政放权政策，对市级现有的行政审批项目依法逐项清理，依法减少和规范行政许可和行政审批。简化保留项目的手续，提高效率。建立并完善与省激励型财政转移支付机制相配套的考核、约束、监督、激励和惩处机制，增强县域经济自主增长的积极性。突出乡镇在县域经济发展中的基础地位，坚持分类指导、差别化竞争、错位发展战略，夯实县域经济的基础，促进县域经济在展现发展共性的同时，彰显发展个性，形成促强扶弱带中间的竞相发展态势。力争所有县(市、区)的财政总收入过2亿元，并形成经济总量超100亿元、财政总收入超10亿元的经济强县。

　　第五，以提高承载水平为重点，继续推进各项基础设施建设。

　　加快交通建设。力争开工建设上武高速公路，配合建成景鹰高速公路；力争上乐公路、湖岑公路和余干龙津大桥、鄱阳古县渡大桥、莲湖大桥建成通车；开工建设上饶经禹溪至省界、弋阳至云际关、婺源清华至吴楚分源、珠曹线、余干中洲大桥等工程；改造和硬化农村公路 1500公里 ；做好三清山（赣东）机场项目前期工作。

　　加快能源建设。开工建设上饶50万伏输变电工程，新建广丰22万伏、鄱阳22万伏输变电工程，开工建设丁家洲、三清山、煌固、石镇和许村等五个11万伏输变电工程；新建龟峰至上饶22万伏供电线路；加快黄金埠电厂二期工程的前期工作进度；继续做好鄱阳小鸣嘴风力发电项目的前期准备工作。

　　加强信息网络建设。由上饶移动公司投资1.6亿元实施信息化三阶段工程、投资1.5亿元推进科技兴农工程；由上饶联通公司投资7000万元建设G网十二期工程；由上饶电信公司继续推进“自然村通电话、行政村通宽带”二期工程，实现90%以上行政村通宽带。

　　（二）着力实施民生工程

　　各位代表，努力解决好广大群众最关心、最直接、最现实的利益问题，是构建和谐社会的内在要求，也是各级政府义不容辞的责任。今年，我们将把实施民生工程作为政府工作的第一项任务，着力实施八大工程，重点抓好40件实事。

　　第一，抓好就业再就业工作，为人民群众解决基本生计和提高生活水平提供重要保证。重点抓好7件实事：新增城镇就业人数4.67万人，城镇净增就业人数4万人，就业率达到94%；新转移农村劳动力8.1万人；新增困难群体就业（“4050”人员就业）人数0.17万人；建立促进“零就业家庭”就业的长效机制，零就业家庭就业安置率达到100%；免费为有创业愿望和创业条件的下岗职工、高校毕业生、城镇复员退伍军人、农民工提供创业培训和创业指导，城镇职业培训人数5.1万人，农村劳动力转移培训人数11.4万人，其中，金蓝领工程7.5万人，阳光工程2.7万人，雨露计划1.3万人；在劳务输出人员相对集中的省外大中型城市，建立健全市级驻外劳务服务管理机构，免费为外出劳务人员提供全方位服务，确保劳动合同签订率达到92%，跨省劳务输出人数达到103.2万人；完善小额贷款激励机制，根据新增小额贷款额度，对100%还贷的给予适当奖励，确保全年小额贷款达到1.1亿元。

　　第二，重视社会保障工作，逐步扩大社会保障的覆盖面，形成经济发展和社会保障相互促进的良好局面。重点抓好2件实事：进一步扩大社会养老保险覆盖面。城镇职工参加基本养老保险人数（含离退休人员）达到45.8万人，农民工参加养老保险3万人，城镇大集体企业职工参加养老保险1万人，工业园区企业职工参加养老保险2万人，被征地农民参加养老保险0.6万人，养老保险参保率达到56%，养老保险基金征缴总额达到5.6亿元，城镇企业离退休人员基本养老金每人每月达到699元；进一步加强医疗保障。城镇职工基本医疗保险参保人数达到40.3万人，参保率达到56.8%，统筹资金使用率达到80%；国有困难企业职工参加医疗保险5.8万人，城镇职工工伤保险参保23.5万人，农民工参加工伤保险3.1万人。

　　第三，强化社会救助工作，逐步建立面向各类特困群众，政策相统一，标准相衔接的城乡一体的社会救助体系。重点抓好6件实事：提高农村低保补差水平，月人均补差达到30元，救助17.3万人；提高城市低保补差水平，低保对象人均补差提高到80元，救助11.86万人；农村五保集中供养率达到80%；精简退职老弱病残职工人均补助150元，对农林水困难企事业单位的0.8万退休职工生活人均补助260元，对参保的1.09万城镇集体企业退休职工人均补助200元；继续改建、扩建、新建城乡敬老院、福利院、光荣院；集中资金实施“蓝天计划”，进一步改善孤残儿童的生存、生活条件。

　　第四，扎实推进各项扶贫工作，加快贫困村脱贫致富、改变面貌的步伐。重点抓好5件实事：坚持自然资源开发和人力资源开发并重，加大培训力度，确保完成万余名贫困农民的劳动力转移培训任务，努力提高劳动者素质；突出抓好以10万亩泡桐为重点的产业化扶贫，努力培育增收产业；继续抓好“十一五”329个省定扶贫重点村的整村推进工作，以及深山区、库区移民扶贫工程，完成搬迁8500人；继续抓好机关单位定点扶贫、干部包户扶贫，努力提高定点扶贫效果；坚持政府主导、社会参与和群众自力更生相结合，继续抓好光彩事业社会化扶贫。

　　第五，加大济困助学力度，促进教育公平。重点抓好6件实事：全部免除全市义务教育阶段公办学校学生学杂费，为全市23万多名贫困家庭学生免费提供教科书，补助6.3万名贫困家庭寄宿学生的生活费，每人每年补助300元；建立农村义务教育阶段中小学校舍维修改造长效机制，各级财政今年安排4876.7万元，修建校舍面积12万平方米；增加义务教育公用经费，农村小学生均公用经费由40元/年增加到74.5元/年，初中由60元/年提高到94.5元/年，城市小学生均公用经费为80元/年，初中为110元/年；建立高中、中职和考取大学的特殊困难家庭学生资助政策体系，从今年起，对公办高中的特殊困难家庭学生每人每年补助800元，补助7410人，对职业高中，普通中专、成人中专、技工学校的特殊困难家庭学生每人每年补助1000元，今年补助6115人，对考取大学的特困家庭学生每人一次性补助5000元，今年补助2470人；进城农民工子女与城镇居民子女一样就近入学，不得加收借读费等不合理费用；确保中小学教师工资按国家标准足额按时发放，教师的各种政策性津贴、补贴不低于当地公务员水平。

　　第六，加快构建公共卫生服务体系，努力解决看病难、看病贵问题。重点抓好7件实事：将新型农村合作医疗试点县扩大到10个，参合县比率达到83%，参合农民达到414.4万人以上，参合率达到85%以上，参合医疗水平每人每年51.7元，筹集合作医疗资金21422.8万元，资金使用率达到75%以上；增加城乡困难群众大病医疗救助资金投入，筹集农村困难群众大病医疗救助资金1198.4万元，其中，市级377.4万元，县级821万元，救助病种12种，救助人数10万人，资金使用率达到80%，筹集城市困难群众大病医疗救助资金969.5万元，其中，市级363.1万元，县级606.4万元，救助人数2万余人，救助病种12种，资金使用率达到80%；建立城镇居民合作医疗制度，城镇居民参加合作医疗人数达到20万人，参合率达到33%，参合医疗水平每人每年50—150元，资金使用率达到80%；对城镇大集体困难企业退休职工和国有农垦、农场、林场、水利困难企事业职工参加城镇合作医疗给予资助；增加投入，改善村卫生所、乡镇卫生院和县级综合医院医疗用房和设备条件，培训医务人员，增强卫生服务能力，争取有645个村卫生室（所）、73个乡镇卫生院、6个县级综合医院、5个县级中医医院达到建设标准，有8个县级疾控机构实验室疾病检测检验能力达到建设标准；增加城市社区基本公共卫生服务经费，扩大城市社区基本公共卫生服务覆盖面，争取有10个社区卫生服务机构达到建设标准；对药品、医疗器械市场和医疗费用进行专项督查整治，重点治理药品价格虚高和医药领域商业贿赂，提高对药品、医疗器械和食品的检验监测能力，保障人民群众的饮食、用药安全。

　　第七，改善群众的生活、生产条件，让群众得到更多的实惠。重点抓好4件实事：有序推进经济适用房建设，全市建设经济适用房50.37万平方米，受益人数达到6297户，其中中心城区建设14万平方米，1750户受益；进一步推进廉租房制度建设，全市实施保障总户数达到1249户，其中中心城区602户；改造和硬化农村公路 1500公里 ，行政村道路硬化率达到62.5%；推广使用新资源，农村使用沼气新增2.5万户。

　　第八，千方百计增加群众收入，努力从多方面提高群众的生活水平。重点抓好3件实事：把提高人民生活水平寓于经济发展之中，在经济发展的基础上，不断增加群众收入，使全体人民都能及时享受到改革开放和社会主义现代化建设的成果。高度重视协调各方面的利益关系，进一步落实好中央和省里的一系列支农惠农政策，减轻农民负担，增加农民收入；提高最低工资标准，增加城镇职工工资性收入；实施好公务员工资制度改革，规范机关事业单位的津贴补贴制度，确保城镇居民人均可支配收入达到10815元，农民人均纯收入达到3760元，在岗职工平均工资达到14310元。

　　为确保“民生工程”的40件实事落实到位，各级政府要用系统的思维去谋划，用抓工程的办法来组织实施。要层层签订责任书，并切实按照责任书的总体目标和实施期限，进一步细化和分解目标任务。要加大对社会保障的支持力度，积极探索好、执行好透明、公开、公平的操作方法和措施，确保这项工作公开公平公正，使符合条件的、真正困难的家庭得到实惠。

　　（三）深入推进改革开放

　　一是协调推进各项改革。深化企业改革。推进国有企业资产重组和企业经营机制转换工作，认真做好医药商业企业整体划转市县管理工作，努力争取和实施朝阳磷矿政策性破产。大力发展资本市场，积极推动企业股份制改革和上市工作，加快市汽运集团等5户企业组建股份公司进程，争取市汽运集团明年上市。深化农村综合改革。开展化解乡村债务试点工作，实施国有农（林）场管理体制改革，继续完善林业产权制度配套改革，深化水权制度改革，完成种子管理体制改革。深化行政管理体制和事业单位人事制度改革。进一步完善行政服务中心和公共资源交易中心功能，认真履行国有资产监管职责，确保国有资产保值增值，积极稳妥地推进事业单位改革。推进财政管理体制改革。抓紧研究制定与省40项公共财政政策相配套的操作方案，确保40项公共财政政策资金足额到位；认真做好行政事业单位工资制度改革和规范公务员津贴补贴工作；认真做好行政事业单位国有资产的登记清理工作。加大“乡财县管”力度，锁定债务，化解风险，确保全市“乡财县管”改革面达到50%，明年全面铺开。

　　二是着力提升外向型经济发展水平。创新招商引资办法。围绕工业、交通、农业、旅游及商贸流通、城建和基础设施、社会事业等重点领域，把招商引资任务分解落实到市、县11个工业园区、三清山管委会和市、县政府分管领导，并实行县（市、区）和有关单位一把手负责制。继续抓好招商引资工作。力争引进10家有影响的国际大企业来饶投资兴业。同时，加快推进春和集团、蓝天集团、六寸晶园、大屏幕液晶、世邦物流园、欧意美鞋业、婺源茶园种植基地开发、荧光化工、丰华纺织、宝鸿鞋业等125个投资5000万元以上的在建和续建项目的建设。积极扩大外贸出口。加大对外贸出口奖励和扶持力度，对出口强劲、潜力大及后发优势明显的企业和出口大户给予重点扶持和贷款担保，帮助企业做强做大。加快上饶海关的建设步伐，搭建良好的外贸平台。深化与各区域间的经济合作。进一步加强与武汉经协区、福州经协区、“9＋2”泛珠三角区域协作区的沟通，精心组织、全力办好“2007赣浙闽皖九方经协区”三届三次党政联席会议。积极参与省里组织的大型招商活动，充分利用“万商西进”活动平台，深入推进开放型经济发展。力争全年实际引进市外资金增长16.2%，达到330亿元；实际利用外资增长18.3％，达到2.2亿美元；外贸出口增长58.1%，达到2.6亿美元。

　　三是深入推进全民创业。全面落实全民创业30条。进一步激活各类创业主体，支持经营管理和科技人员领头创业，吸引外出务工经商人员返乡创业，大力扶持下岗失业人员自主创业，鼓励高校毕业生自立创业，推动农民致富创业，引进人才来饶创业，着力营造“百姓创家业、能人创企业、干部创事业”的创业氛围。不断健全社会化服务体系。探索建立中小企业联保制度，帮助企业解决资金短缺等实际困难；继续发挥工商联、行业协会、商会等组织的作用，促进民营企业健康发展。力争全年净增个体工商户5000户，净增私营企业1000家，净增个私企业注册资本金21亿元，总量达到176亿元。

　　（四）进一步加快社会事业发展

　　一是抓好科技成果转化。强化科研攻关,开展多种形式的产学研联合,搞好重点产业中关键技术的引进、消化、吸收,加快科技成果向现实生产力转化，努力形成以自主创新为动力的增长结构，以科技创新型产业为主导的产业结构，以自主创新型企业为核心的企业结构，争取建立1至2家省级企业技术中心、3个上级技术创新扶持项目。

　　二是切实把教育放在优先发展的战略地位。把扶持农村义务教育放在更加突出的位置，促进城乡义务教育均衡发展，确保今年完成农村寄宿制学校工程建设任务，在48所学校实施“新农村卫生新校园建设工程”，加快实施农村中小学现代远程教育工程，确保今年全面达标。大力发展职业教育，加强技能型人才培养。做大做强上饶职业技术学院，努力扩大办学规模，提高办学质量，加强鄱阳职教中心、玉山职业中专等一批品牌职校建设，今年建设2所省级示范性职业学校，重点建设5个职业教育实训基地，确保中等职业教育招生规模增长25%以l。继续鼓励和规范社会力量办学，促进民办教育健康发展。高度重视成人教育、继续教育，倡导终身教育。强化学校安全稳定意识，促进和谐校园建设。

　　三是全面加强人口与计划生育工作。坚持计划生育基本国策和稳定现行计划生育政策不动摇；党政一把手亲自抓、负总责不动摇；稳定人口和计划生育工作机构、队伍，创新工作体制、机制、手段和方法不动摇；实行计划生育“一票否决”不动摇。全面实行农村计划生育家庭奖励扶助制度，稳定低生育水平,防止人口出生率反弹，有效降低出生人口性别比。

　　四是加快文广卫体等事业发展。加快市文化艺术中心、信江书院、影剧院等文化设施项目建设，抓好县图书馆、文化馆和乡镇文化站建设，不断丰富群众文化生活。加强文化市场管理，深入开展“扫黄打非”，规范网吧、音像等行业经营。加快广电传输网络改造项目建设，确保今年乡镇光缆联网率达到95%。加快启动有线电视数字化整体转换工程。强化公共卫生职能。加强疾病预防控制和医疗救治体系建设,加快疾控中心实验室标准化建设，提高处理重大疫情和公共卫生突发事件的应急处置能力。优化卫生资源配置，健全城市社区卫生、农村卫生服务体系。抓好“农民体育健身工程”建设。今年全市争取完成60-80个农民体育健身工程项目，做好体育后备人才的选择、培养工作，促进竞技体育、群众体育协调发展。重视和支持工会、共青团、妇联等群众性团体工作，关心妇女、儿童、老龄事业，积极发展残疾人事业和慈善事业，认真做好民族、宗教、对台、外事、侨务、防震减灾、人防、气象、档案、新闻出版、保密和地方志等工作。

　　五是加强平安上饶建设。继续推进“食品药品放心工程”，加强粮食、食品、药品和饮用水源监管，切实保障人民群众健康安全。加强对煤矿、非煤矿山、交通、危险化学品和森林防火等重点领域安全工作，坚决遏制重大安全事故发生。进一步健全和完善社会治安防控体系，不断加强社会治安综合治理工作，强化重点领域、重点环节、重点区域的治安巡防，加大对刑事犯罪的打击力度，创造平安祥和的社会治安环境。完善信访和人民调解工作，加强市长热线和应急管理工作，推进普法教育、法律服务和行政复议工作，加大化解各种矛盾纠纷的力度，着力解决群众反映强烈的问题，深化和谐平安创建，切实维护社会稳定。重视国家安全工作，进一步加强民兵预备役和国防动员工作。

　　（五）切实抓好资源节约和生态保护

　　一是加强资源节约和综合利用。大力推进节能。突出抓好高耗能企业节能，重点抓好房屋建筑土地(小区)节能试点和政府建筑物、办公系统节能改造；正确引导商业和民用节能。大力推进节材和循环经济，推动节能节材和资源再利用。着力抓好15家资源综合利用企业、1家清洁生产企业、16家再生资源回收和利用企业等循环经济典型企业。认真落实各级政府土地管理和耕地保护责任制，建立规范有序的市县乡村和农民用地管理、督查和处理机制，加强对基本农田保护区的保护。组织实施好土地开发整理项目，保质保量补充耕地，稳定全市耕地总量。抓好矿产资源的合理开发和综合利用。加强对矿产资源勘查、开采、使用的有序管理，鼓励符合国家产业政策、符合省重点支持产品目录的项目发展，鼓励社会资金投入商业性地质勘查，促进矿产资源开发由掠夺型向综合利用、规模效益型转变。

　　二是严格控制污染物排放。实行环境保护行政负责制和“环保一票否决”制。严把环境保护准入关，切实做到“三个坚决不搞”；严格遵守环境保护设施与主体工程“三同时”制度，未执行“三同时”和未经环保验收的项目不得投产。实行严格的排污许可制度。把二氧化硫和化学需氧量总量控制指标分解落实到各级政府，确保到2010年底实现全市化学需氧排放量比2005年减少7.5%，由3.99万吨削减到3.69万吨，二氧化硫排放量控制在4.46万吨以内。对超过污染物排放总量控制指标、生态破坏严重的地区，停止审批新增污染物排放的建设项目；对污染处理设施不能稳定达标或超总量排污的企业，坚决责令整改，直至关闭。切实保护好饮用水源安全。进一步加强水源地污染治理力度，限期治理信江河上饶城区段及其上游的工业污染企业，上饶城区段一江两岸的排污口要在年内治理完毕，沿河上下游排污口也要列入治理计划，不能治理或治理后仍不能达标排放的企业要一律关闭。积极推进循环经济。加快推行清洁生产，扎实推进畜禽养殖场点的污染综合治理，建设一批生态农业示范工程。各县（市）要尽快开工建设污水处理厂，对生活污水进行集中处理。完成县城水源保护区划定方案的编制和审批工作，加强建制镇的水源保护，并着手保护区的划定工作。

　　三是全面推进生态环境建设。坚持经济发展与生态保护相统一，努力建设绿色生态上饶。大力推进生态公益林建设，坚决落实好生态公益林的补偿政策，今年新增中央森林生态效益补偿面积175万亩，省地方生态公益林补偿面积122.3万亩。严格控制森林特别是天然阔叶林的采伐，严厉打击乱砍、滥伐、偷运、偷伐。抓好植树造林和沿江、沿湖、沿路绿化工作，构筑城乡绿化体系，全年完成植树造林面积50万亩。认真实施信江上游水土治理项目和国家农业综合开发水土保持项目，继续抓好信江、乐安河上游水质保护和水土保持。进一步加强鄱阳湖湿地保护，维护湿地生态功能和生物多样性。

　　三、进一步加强政府自身建设

　　各位代表，新的形势和任务对政府工作提出了更高标准和要求。我们将全面加强政府自身建设，努力建设法治政府、服务政府、责任政府和效能政府，提高政府的执行力和公信力。

　　一是加强民主法制建设。认真执行人大及其常委会的各项决议，不断完善工作报告制度，依法自觉接受监督；主动听取人民政协的意见和建议，积极支持政协履行政治协商、民主监督、参政议政的职能；切实做好人大代表的议案、建议和政协提案；广泛听取各民主党派、工商联、无党派人士和人民团体的意见、建议；继续深入贯彻行政许可法和依法行政纲要，严格按照法定权限和程序履行职责，进一步提高依法行政的能力和水平。

　　二是转变政府职能。改进政府管理方式和服务方式。积极推进政企分开，规范发展社会中介组织，着力解决政府职能缺位、错位、越位的问题，坚决把不该由政府管理的事交给市场、企业、社会组织和中介机构。着力提高行政效能。继续强化市、县行政服务中心功能，加大依法清理行政许可事项力度，把该减的减到位，该清的清到底，努力推进并联审批，提高行政效率和服务水平。不断强化政府的社会管理和公共服务职能。把财力等公共资源更多地投向社会管理和公共服务，着力解决民生问题，努力为广大人民群众提供更多的公共服务产品，促进社会全面发展。

　　三是推进政务公开。建立和完善群众参与、专家论证和政府决策相结合的决策机制。对涉及群众利益的重大问题，实行社会公示和专家咨询听证；完善新闻发布制度。继续办好市长热线和政府网站，加快电子政务建设，及时公布老百姓关心的事，努力办好老百姓希望办的事；健全和完善行政执法依据、程序公开制度，着力营造公开、公正、规范、便捷的政务环境。

　　四是加强政风建设。按照市委、市政府的统一部署，切实搞好“加强作风建设，实现新的跨越”集中教育活动，深入推进政风、行风、机关作风“三风”建设，进一步树立大局观念、增强宗旨意识、强化进取意识，不断健全和完善目标管理及重大事项督办制度，促进各项工作落实。切实加强公务员队伍的教育、培训、管理和监督，提高公务员政治、业务素质。认真纠正和克服本位主义、形式主义、享乐主义和无为思想等问题，始终保持奋发有为、昂扬向上的精神状态，努力形成求真务实、政令畅通的工作局面，着力提高各级政府的理解力、操作力和执行力。

　　五是推进廉政建设。认真执行党风廉政建设的各项纪律，自觉按照法律和制度的规定办事。不断强化行政监察和审计工作，加大查办商业贿赂案件力度，重点治理工程建设、土地出让、产权交易、医药购销和政府采购等领域的商业贿赂问题，坚决纠正和查处不正当交易行为。进一步规范公务接待活动，提倡勤俭节约，弘扬艰苦创业，反对奢侈浪费，堵塞管理漏洞，促进廉洁从政，努力建设廉洁政府。

　　各位代表，上饶正处在又好又快发展的关键时期，我们的任务非常艰巨，责任极其重大，使命无上光荣。让我们高举邓小平理论和“三个代表”重要思想伟大旗帜，在中共上饶市委的领导下，以科学发展观统领经济社会发展全局，团结拼搏，开拓进取，扎实工作，全面完成2007年各项任务，为建设富强民主文明和谐的新上饶而努力奋斗！
